# Role of Cone-Beam CT in the Intraprocedural Evaluation of Chemoembolization of Hepatocellular Carcinoma

**DOI:** 10.1155/2021/8856998

**Published:** 2021-03-19

**Authors:** Antonio Orlacchio, Silvia Roma, Vito dell'Olio, Sara Crociati, Ilaria Lenci, Simona Francioso

**Affiliations:** ^1^Department of Diagnostic and Interventional Radiology, University Hospital Tor Vergata, Viale Oxford 81, 00133 Rome, Italy; ^2^Liver Unit, University Hospital Tor Vergata, Viale Oxford 81, 00133 Rome, Italy

## Abstract

**Purpose:**

To assess the ability of Cone-Beam CT (CBCT), performed during the Transcatheter Arterial Chemoembolization (TACE), in predicting the response to treatment of hepatocellular carcinoma (HCC).

**Materials and Methods:**

We evaluated fifty patients (M/F = 40/10, mean age: 66.7 years ± 8.22) with hepatocellular carcinoma (HCC), for a total of 82 nodules evaluated (mean diameter: 21.4 ± 11 mm). All patients performed a CT scan one month before and one month after TACE. After TACE is completed, a CBCT was performed to assess the degree of drug retention in the lesions. For each lesion, the major diameter, volume, and density of the vital portion were evaluated. The response to TACE was assessed using the mRECIST criteria on the CT scan carried out one month after the procedure. The receiver operating characteristic (ROC) curves were performed to assess the accuracy of the CBCT in predicting the response to treatment and to identify the cut-off points for each parameter under examination.

**Results:**

A complete response (CR) was observed in 24/50 patients (48%), a partial response (PR) in 16/50 (32%), stable disease (SD) in 8/50 (16%), and progressive disease (PD) in 2/50 (4%). Evaluation of the area under the ROC curve showed that the diameter, volume, and density of the lesion, measured with CBTC, had an accuracy of 94%, 96%, and 98%, respectively, in discriminating a complete response from a not complete response.

**Conclusion:**

CBCT is effective in predicting short-term response at 1-month follow-up of HCC treated by chemoembolization.

## 1. Introduction

Hepatocellular carcinoma (HCC) is the principal malignant liver cancer (90%) and the second cause of cancer-related death worldwide [1]; most patients come to the diagnosis of HCC in the intermediate/advanced stage [2].

The EASL guidelines recommend treatment with Transcatheter Arterial Chemoembolization (TACE) in intermediate stage HCC [1]. In these patients with multinodular, unresectable, and nonmetastatic HCC, the average survival is 16 months, which may increase to 40 months after TACE [3].

The response to treatment is usually evaluated 1–3 months after the procedure with Computed Tomography (CT) or Magnetic Resonance Imaging (MRI) through modified Response Evaluation Criteria in Solid Tumors (mRECIST) guidelines [4].

Intraprocedural Cone-Beam CT (CBCT) has been recognized to be an effective and valuable tool for detection of enhancing portion in many interventional procedures including TACE [5–10], so it could be useful for detection of remnant viable tumor after TACE.

The purpose of this study is to evaluate the ability of CBCT, performed at the end and sometime during Degradable Starch Microspheres Transcatheter Arterial Chemoembolization (DSM-TACE), in predicting tumor response in patients with unresectable HCC.

## 2. Methods

### 2.1. Study Cohort

The single-center retrospective study protocol was approved by the ethical institutional committee. Informed consent was obtained before TACE from all patients.

From September 2018 to June 2019, 149 patients with unresectable HCC and who were treated with TACE were evaluated.

Diagnosis of HCC was carried out conforming to the guidelines of the European Association for the Study of the Liver (EASL) [1].

The choice of treatment was made through the evaluation of a multidisciplinary team (MDT) of hepatologists, surgeons, and interventional radiologists.

Eligibility criteria for DSM-TACE were as follows: focal or multifocal unresectable HCC, Child-Pugh classification A or B, Eastern Cooperative Oncology Group performance status 0 or 1, and no contraindication to contrast medium.

Patients with extrahepatic disease, complete neoplastic thrombosis of the portal vein, values of bilirubinemia >3 g/dl, and creatinine levels >2.0 mg/dL were excluded.

Target lesions were admitted to images' analysis through the following eligibility criteria:

1. Contrast-enhanced dynamic multislice CT (CECT) acquired within 30 days or less before and 30 days ± 2 days after TACE.
2. HCC treated with DSM-TACE.
3. Intraprocedural CBCT acquired immediately after TACE. When re-TACE was necessary, we repeated CBCT at the end of procedure.
4. Target lesion visualized without artifacts on CT and CBCT.
5. Well-defined tumor borders.

Patients and lesions that did not meet the aforementioned criteria were not included in our study.

Ninety-nine patients were excluded (missing CBCT: 54/149; MRI evaluation: 13/149; Drug-Eluting-Beads-TACE: 13/149; Lipiodol-TACE: 9/149; missing one month before procedural CT: 10/149).

Fifty patients without resectable HCC were selected (Table 1), for a total of 82 HCC lesions evaluated (mean diameter: 21.4 ± 11 mm).

Of these patients, 24 had stage A (early) and 26 had stage B (intermediate) HCC of the Barcelona Clinic Liver Cancer (BCLC) staging system [11].

### 2.2. CT Protocol

CECT examination was performed one month before TACE using a 64-slice CT scanner (Lightspeed; General Electric Healthcare, Waukesha, WI). The scanning protocol was as follows: 0.6-second rotation time, pitch 0.9, 120 kV, 250 mA, and image thickness of 2.50 mm. The CT examination consisted of an unenhanced CT scan of the liver and, after intravenous administration of contrast medium (Iomeprol 350 mgI/mL; volume: 100–110 mL; flow rate: 3 mL/s), an arterial phase at 35 s, and a portal and delayed phase at 60 s and 120 s from the beginning of contrast media administration, respectively.

Follow-up CT scan was performed, with the same protocol, one month after TACE to evaluate the tumor response.

### 2.3. DSM-TACE Protocol

All procedures were performed in a dedicated angiography suite room (Allura Xper FD20, Philips Healthcare) equipped with the XperCT option, enabling CBCT acquisition and volumetric image reconstruction.

All patients received a premedication consisting of a proton-pump inhibitor, a prokinetic drug, and an analgesic drug; if necessary, a conscious sedation was performed during procedures.

A preliminary arteriographic examination was performed to evaluate the vascular anatomy and the feeding arteries of HCC and, when useful, a segmental or subsegmental approach was executed using a coaxial 2.7 Fr microcatheter.

Six milliliters of nonionic contrast media were mixed with 4 mL of DSMs (EmboCept S DSM 50/*μ*m—PharmaCept, Berlin, Germany) prior to the injection. Doxorubicin at a dose of 50 mg/m^2^ was diluted in 5 mL of normal saline solution. Thus, a suspension of DSMs, contrast medium, and Doxorubicin was obtained for endovascular administration. The mixture in the syringes was constantly agitated and slowly injected at the proper site until initial stasis of flow was observed on tumor feeding vessels. At the end of mixture of DSMs, Doxorubicin, and contrast medium, DSMs alone was slowly and continuously injected until a complete embolization was obtained.

Immediately after the DSM-TACE, unenhanced CBCT scan was acquired to assess deposition in hepatocellular carcinoma of the mixture.

When, at CBCT examination, complete chemoembolization was not achieved, immediate re-TACE within the same session was performed in about ten percent of cases. In our study, we considered data from the final CBCT examination.

### 2.4. Intraprocedural C-Arm Cone-Beam CT Protocol

CBCT was performed using the same angiographic system with the XperCT option, enabling CBCT acquisition and volumetric image reconstruction.

Patients were instructed to hold an end-expiratory apnea during the CBCT scan. In 10 seconds, 312 projection images (60 frames per second) were acquired, while the C-arm rotates covering a 180° clockwise arc under a fixed 123 kVp and 325 mAs setting.

The resulting two dimensional projection images were immediately reconstructed using Feldkamp back projection into 3D volumetric images for a 250 × 250 × 194 mm field of view (matrix size 384 × 384 × 296) with a voxel size of 0.6 mm^3^.

The dose exposure for one CBCT abdomen scan is approximately 3–10 mSv.

### 2.5. Imaging Evaluation

On each lesion, the largest diameter, volume, and CT density were measured in the CT scan performed within one month before the procedure. The volume of the lesions was calculated, using the semiautomatic segmentation system in Livewire mode (Carestream, Rochester, NY). The density was evaluated by inserting ROIs at different levels on the whole portion of lesion enhanced. Three density measurements were made and the average density value obtained was recorded.

In the second instance, the images obtained with the intraprocedural CBCT were analyzed with the same methodology as that for preprocedural CT. Also in these cases the largest diameter, volume, and density of the chemoembolization mixture retained in the lesions were evaluated. Hounsfield units were calculated in CBCT using the formula proposed by Mah et al. [12].

The diameter and volume obtained from the CBCT were compared with the same data obtained in the preprocedural CT and the percentage of drug retention was calculated for each lesion with the same methodology as that for preprocedural CT.

The HCC response to TACE was evaluated through the application of the mRECIST criteria in the CT scan performed 30 days after the treatment.

### 2.6. Statistical Analysis

On each lesion, average diameter (mm), volume (mm³), and density (HU) were evaluated.

Furthermore, the drug retention percentage of the lesions evaluated on CBCT was calculated on the enhancement areas evaluated on preprocedural CT for both diameter and volume values.

Resulting data were divided into two groups [complete response (CR) and not complete response (nCR)] based on the tumor response evaluated on one month after procedural CT scan according to the mRECIST.

Data were expressed as mean and standard deviation (SD).

For each parameter, the analysis of variance between the two groups was carried out using Analysis of Variance (ANOVA).

To determine the CBCT accuracy in predicting HCC response to DSMs-TACE and the appropriate cut-off points of every parameter, the receiver operating characteristic (ROC) curves were developed. For ROC curve analysis, the area under the curve (AUC), 95% confidence intervals (CI), the optimal cut-off value, sensitivity, specificity, positive predictive value, and negative predictive value were computed.

Statistical significance was set at *p* < 0.05.

All statistical analyses were performed with IBM SPSS Statistics software (release 15; IBM, Armonk, NY, USA).

## 3. Results

**Figure 1:**
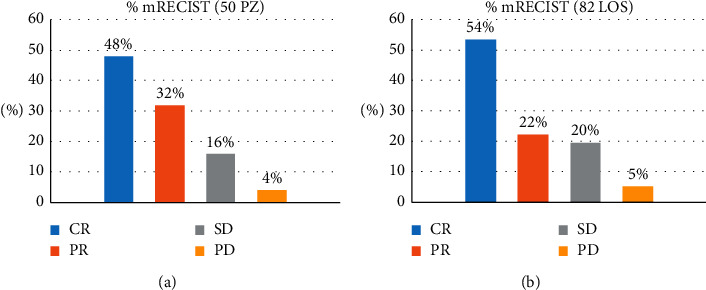
Response rate according to mRECIST criteria in 50 Patients (a) and in 82 lesions (b). CR: complete response; PR: partial response; SD: stable disease; PD: progressive disease.

In accordance with mRECIST criteria, a complete response (CR) was obtained in 24/50 patients (48%), partial response (PR) in 16/50 (32%), stable disease (SD) in 8/50 (16%), and progressive disease (PD) in 2/50 (4%) (Figure 1(a)).

Furthermore, we individually evaluated the response of the 82 HCC lesions, following the mRECIST criteria. A CR was achieved in 44/82 (53.6%) lesions and a PR in 18/82 (21.9%) lesions, while SD was noted in 16/82 (19.5%) and PD was noted in 4 (4.9%) lesions (Figure 1(b)).

**Figure 2:**
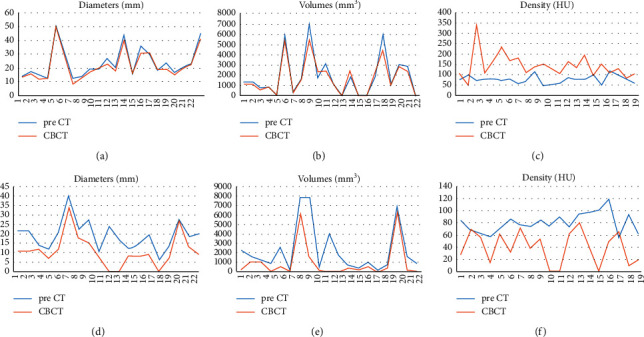
Trend of values of diameters, volumes, and density in lesions with complete response (a–c) and with not complete response (d–f) in preprocedural CT (pre-CT) and in the Cone-beam CT (CBCT) performed during TACE.

**Figure 3:**
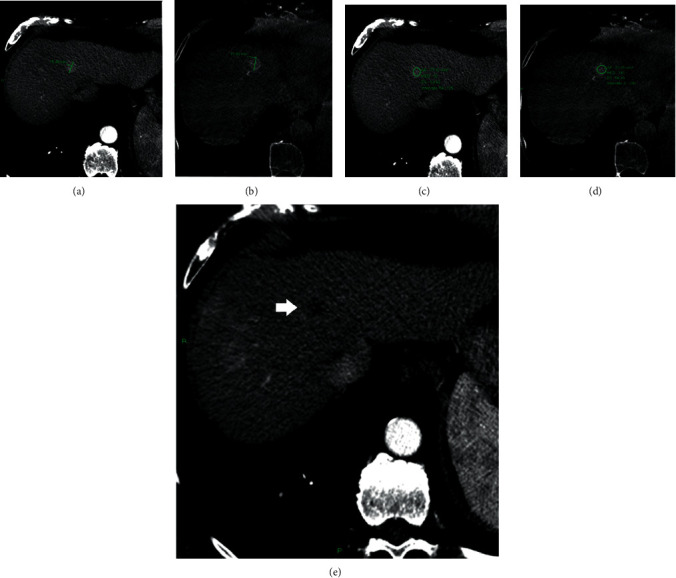
Diameter and density of an HCC nodule on the preprocedural CT (a, c) and on the CBCT (b, d). Contrast enhancement CT at 1 month from the procedure shows complete response to the DSM-TACE (e).

In the group of lesions with CR, the mean value of diameter, volume, and density on the preprocedural CT were 23.71 ± 10.99 mm, 1924.5 ± 2022.69 mm³, and 77.14 ± 19.35 HU, respectively, and 21.98 ± 10.61 mm, 1726.27 ± 1677.69 mm³, and 144 ± 59.75 HU on CBCT (Figures 2(a)–2(c) and 3). In this group, at one month ±2 days, all lesions showed no enhancing areas on the follow-up CT examination.

**Figure 4:**
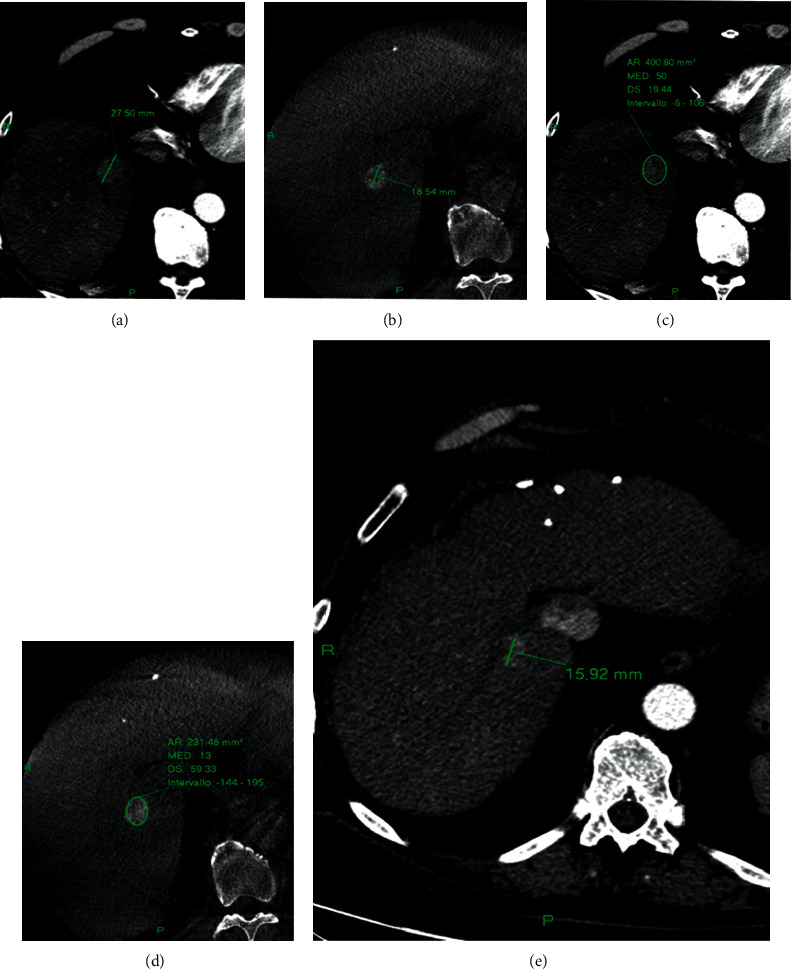
Diameter and density of an HCC nodule on the preprocedural CT (a, c) and on the CBCT (b, d). Contrast enhancement CT at 1 month from the procedure shows not complete response to the DSM-TACE (e).

In nCR lesions, the mean value of diameter, volume, and density on the preprocedural CT were 19.19 ± 7.69 mm, 2275.7 ± 2543.53 mm³, and 80.74 ± 16.72 HU, respectively, and 11.09 ± 8.44 mm, 982.28 ± 1883.89 mm³, and 39.84 ± 26.66 HU on CBCT (Figures 2(d)–2(f) and 4). At one month, these lesions still presented a viable enhancing tumor area (mean diameter 14.23 ± 5 mm, mean density 79.34 ± 29.26 HU) on the follow-up CT.

The amount of chemoembolization mixture retention of the lesions was evaluated on CBCT by measuring the largest diameter of tumor enhancing and by calculating its volume. The percentage results were equal to 91.7%, 87.3%, and 205.4% of the baseline CT parameters, respectively, in the 44 CR lesions. Instead, it was much lower in nCR lesions and equal to 54.7% of the preprocedural diameter and 36.6% of the preprocedural volume and average 52.4% over the tumor baseline density.

The statistical analysis showed a statistically significant difference (*p* < 0.05) between the two groups (CR and nCR) of the evaluated parameter values (largest diameter, volume, and density on CR CBCT and nCR CBCT examinations distribution. CR lesions' Odds Ratio and Hazard Risk of main diameters: 24.75 and 2.98; Odds Ratio and Hazard Risk of CR lesions volumes: 15 and 2.27).

**Figure 5:**
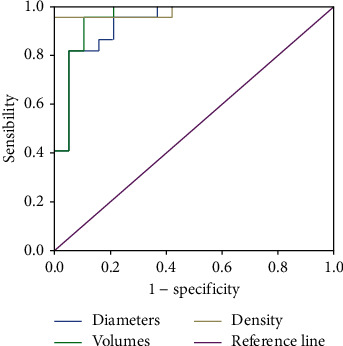
Receiver operating characteristic (ROC) curves of diameters, volumes, and density.

The evaluation of the area under the receiver operating characteristic curve (AUC) showed that high percentage value of the largest diameter and the volume and density in terms of amount of chemoembolization mixture retention of the lesion evaluated at CBCT presented accuracies of 94%, 96%, and 98%, respectively, to discriminate CR from nCR (Figure 5).

In particular, the CBCT finding of a diameter of the chemoembolization mixture retention area of 85.91% of the preprocedural diameter has a positive predictive value of 94.7% response to treatment (Table 2).

Finally, the evidence of a density value of at least 82.5 HU on CBCT is a 100% positive predictive value of treatment response in DSM-TACE.

## 4. Discussion

Our retrospective study underlines the usefulness of intraprocedural CBCT scan evaluation of HCC treated with TACE.

As reported in previous similar studies [13, 14], a strong association between the degree of chemoembolization mixture deposition and nonenhancing necrotic tumor tissue on CT performed at 30-day follow-up is shown. Furthermore, it is demonstrated that quantitative evaluation of chemoembolization mixture deposition on intraprocedural CBCT can predict short-term response at 30-day follow-up CT after DSM-TACE in patients with HCC.

Tumor response is the most important predictive factor of survival. Early assessment of response to treatment is crucial to guide patient management for potential TACE repetition or to add other treatments after chemoembolization.

To determine the tumor response, radiology has a key role through the application of contrast-enhanced imaging techniques like contrast-enhanced CT or contrast-enhanced MRI, which allow detecting the viable tumor tissue as well as assessing the response to TACE treatments making proper therapeutic choices in a short time [15].

Our study illustrates a strong correlation between the grade of mixture retention after chemoembolization, in terms of both quantity and quality, and short-term tumor response on CECT at one-month follow-up. This can allow extending the CBCT field of application to the intraprocedural monitoring and to the assessment of tumor response to TACE.

A CR, in fact, was observed in lesions in which the chemoembolization mixture retention measured on CBCT had nearly the same diameter or volume value as that of the enhancing portion evaluated on preprocedural CT and the density value was higher than that measured in the preprocedural CT.

These results are further strengthened by the evidence on CBCT of lower chemoembolization mixture retention in terms of dimension, volume, and density compared to the vital portion of the lesions previously evaluated on preprocedural CT in lesions with a nCR.

Additionally, our results show that density values of the chemoembolization mixture retention evaluated on CBCT >82.5 HU and >200% in percentage of the baseline density value (HU) of tumor enhancing have a positive predictive value of 100% in case of treatment complete response. However, this result is effective only for our TACE method.

Our findings may drive a change in patients' treatment management if treated lesions still have residual viable tumor tissue after DSM-TACE. Possible therapeutic options might have a shortened retreatment interval or a change of therapeutic choice in patients with unsatisfactory drug deposition assessed at intraprocedural CBCT [16].

Compared to fluoroscopy, CBCT provides an immediate three-dimensional chemoembolization mixture deposition overcoming the limit of 2D fluoroscopy images [17].

The CBCT ability to evaluate intratumoral chemoembolization mixture deposition during TACE procedure allows changing the end-point drug administration by significantly increasing the treatment efficacy [18].

We agree with Kim et al. on the role and utility of CBCT to evaluate technical success after TACE by visual assessment of the results of treatment [19].

Although intraprocedural CBCT for detection of chemoembolization mixture deposition adds more cumulative dose area product (DAP) to the procedure [20], CBCT results in lower overall radiant exposure in TACE; in fact, the use of CBCT during TACE reduces the cumulative dose compared to a procedure using fluoroscopy and digital subtraction angiography (DSA), facilitating the procedure and potentially reducing the procedure time [13].

This study presents several potential limitations, such as the small sample size (50 patients; 82 lesions) and being a single-center, retrospective study. Only “measurable” HCC lesions were included in our study (clear borders and not diffuse/infiltrative disease).

Histopathological examination information was not present after TACE, so no histopathological correlation with imaging was evaluated as concerns the degree of tumoral necrosis after treatment; this was only evaluated on CECT and not on surgical specimens.

Moreover, CBCT reconstructed images were more susceptible to artifacts due to noise, scatter, partial volume effects, and motion artifacts. This may have given a partially careful visual estimation of the tumor spreading, especially in small HCCs.

Another limitation of our study is the absence of the comparison of the parameters evaluated with a control cohort. Further prospective studies should be done on a larger case series including a control group.

Further studies with a larger size sample and that even consider diffuse/infiltrative disease should be planned to confirm our data in the future.

## Figures and Tables

**Table 1:** Baseline characteristics of patients with HCC. AFP: alfa fetoprotein; ALT: alanine transaminase; AST: aspartate aminotransferase; GGT: gamma-glutamyl-transpeptidase; HCV: hepatitis C virus; HBV: hepatitis B virus; INR: international normalized ratio; MELD: model for end-stage liver disease; PLT: platelets.

Characteristics	Absolute value or mean ± standard deviation
Sex (male/female)	40/10
Age	66.7 ± 8.22
Total bilirubin (mg/dl)	1.28 ± 0.6
Creatinine (mg/dl)	0.92 ± 0.25
INR	1.1 ± 0.2
PLT (10^3^/*μ*l)	116200 ± 59505.6
MELD	10 ± 2.73
Child-Pugh, *n* (%)	
A	44 (88%)
B	6 (12%)
Aetiology	
HCV	24 (48%)
HBV	4 (8%)
Alcohol	8 (16%)
Steatohepatitis	12 (24%)
Other (hemochromatosis)	2 (4%)
Previous HCC treatment, *n* (%)	44 (88%)
HCC side (monolobar versus bilobar)	24 vs. 26 (48% vs. 52%)
Nodules number for patient	1.64 ± 0.79
Nodules diameter (mm)	21.4 ± 11

**Table 2:** Receiver operating characteristic (ROC) curves. AUC: area under curve; C.I.: confidence interval; PPV: positive predictive value; NPV: negative predictive value; CBCT: Cone-Beam computed tomography; HU: Hounsfield units.

	AUC	C.I. 95 %	Cut-off HU	Sensibility %	Specificity %	PPV %	NPV %
Main diameters, CBCT versus baseline CT (mm)	0.935	0.861–1	85.91	81.8	94.7	94.7	81.8
Volume, CBCT versus baseline CT (mm^3^)	0.955	0.889–1	73.55	95.5	89.5	91.3	94.4
Density, CBCT versus baseline CT (HU)	0.981	0.942–1	82.5	95.5	100	100	95

## Data Availability

The data are available upon request to the corresponding author.
